# A Systems Biology Approach to Understanding the Mechanisms of Action of an Alternative Anticancer Compound in Comparison to Cisplatin

**DOI:** 10.3390/proteomes2040501

**Published:** 2014-11-10

**Authors:** Elise P. Wright, Matthew P. Padula, Vincent J. Higgins, Janice R. Aldrich-Wright, Jens R. Coorssen

**Affiliations:** 1 Molecular Physiology Department, and the Molecular Medicine Research Group, School of Medicine, University of Western Sydney, Campbelltown, NSW 2751, Australia; E-Mails: e.wright@uws.edu.au (E.P.W.); j.aldrich-wright@uws.edu.au (J.R.A.-W.); 2 Proteomics Core Facility, Faculty of Science, University of Technology, Sydney, NSW 2007, Australia; E-Mail: matthew.padula@uts.edu.au; 3 School of Biotechnology and Biomolecular Sciences, University of New South Wales, Kensington, NSW 2052, Australia; E-Mail: v.higgins@unsw.edu.au; 4 School of Science and Health, University of Western Sydney, Campbelltown, NSW 2751, Australia

**Keywords:** yeast, platinum, proteomics, 2D electrophoresis

## Abstract

Many clinically available anticancer compounds are designed to target DNA. This commonality of action often yields overlapping cellular response mechanisms and can thus detract from drug efficacy. New compounds are required to overcome resistance mechanisms that effectively neutralise compounds like cisplatin and those with similar chemical structures. Studies have shown that 56MESS is a novel compound which, unlike cisplatin, does not covalently bind to DNA, but is more toxic to many cell lines and active against cisplatin-resistant cells. Furthermore, a transcriptional study of 56MESS in yeast has implicated iron and copper metabolism as well as the general yeast stress response following challenge with 56MESS. Beyond this, the cytotoxicity of 56MESS remains largely uncharacterised. Here, yeast was used as a model system to facilitate a systems-level comparison between 56MESS and cisplatin. Preliminary experiments indicated that higher concentrations than seen in similar studies be used. Although a DNA interaction with 56MESS had been theorized, this work indicated that an effect on protein synthesis/ degradation was also implicated in the mechanism(s) of action of this novel anticancer compound. In contrast to cisplatin, the different mechanisms of action that are indicated for 56MESS suggest that this compound could overcome cisplatin resistance either as a stand-alone treatment or a synergistic component of therapeutics.

## 1. Introduction

Platinum-based drugs are among the most commonly and effectively used compounds in the treatment of solid tumours [1,2]. Cisplatin has been used extensively in the treatment of cancers such as head and neck, ovarian, testicular, and small cell lung variants [2,3]. While cisplatin and its derivatives all covalently bind to DNA at purine bases forming intra- and inter-strand crosslinks [4], the differential activities of these compounds arise from their structural differences. The chloride [5], cyclobutane-1,1-dicarboxylate and oxalate groups influence the anticancer potential, range of activity, capacity for cell recognition, and response to the DNA adducts formed [6]. Most cisplatin derivatives are less toxic than the parent molecule and can be used at higher dosages for longer periods [7]. Nonetheless, these compounds produce severe side effects, the most common being nephrotoxicity, ototoxicity and neurotoxicity [1,6]. Moreover, despite the large number of compounds that are structurally similar to cisplatin (e.g., oxaliplatin, nedaplatin), between 1990 and 2002, only 71 new compounds for the treatment of cancer and symptomatic illnesses were found suitable to be granted marketing approval and cleared for clinical use [8]. Among these new molecules were anti-metabolite, retinoid and anti-mitotic anthracycline compounds. Additional platinum based compounds, satraplatin and picoplatin, have also been under trial but are yet to be approved [9,10].

The disparity between supply and demand of viable cancer therapeutics combined with the increasing prevalence of tumour resistance to treatment, drive novel anti-cancer compound formulation and the necessary characterisation of their molecular mechanisms of action. Given the complexity of biological systems and their interaction with chemotherapeutics, it is necessary to apply methods that can accommodate the intricacies of multifaceted responses but also determine mechanisms of action on a system-wide basis [11]. Systems Biology takes the multiple levels of biological hierarchy into account and capitalises on their interrelationships [12]. It is thus generally held, that using such larger-scale approaches (*i.e.*, the ‘Omics’) provides the best access to the fundamental principles of a biological response, in this case to chemotherapeutics.

An alternative mechanism of action for chemotherapeutics that has been investigated is intercalation [13]. The insertion of a compound between the base pairs of DNA results in a longer, partially unwound, irregular helix and this can alter gene expression, replication, cell growth, and cause apoptosis [14,15]. Intercalators previously implemented in chemotherapy include the anthracyclines, duanomycin and doxorubicin, actinomycin D, and the podophyllotoxin, etoposide [16]. An example of a novel intercalator is [(5,6-dimethyl-1,10-phenanthroline)(1S,2S-diaminocyclohexane) platinum(II)]^2+^ (56MESS) [13,17], which has an IC50 of 9 ± 2 nM in L1210 murine leukaemia cells, compared to 500–1000 nM for cisplatin [18,19]. Furthermore, 56MESS has also proven active in cisplatin-resistant cell lines [13]. The molecular basis for this cytotoxicity is a prime subject for investigation, given that this represents a rationally designed alternative therapeutic for overcoming cisplatin resistance in tumours.

Previous studies have indicated that 56MESS does indeed interact with cells via a different mechanism to cisplatin. Microprobe synchrotron radiation X-ray fluorescence (SRXRF) imaging was used to map cellular targets of cisplatin and 56MESS in A549 human lung cancer cells and it was observed that the platinum in 56MESS was primarily localised to the nucleus while cisplatin exposure resulted in more widespread cellular distribution [20]. Moreover, platinum from 56MESS accumulated in the nucleus after only 4 h of exposure. Co-localisation with phosphorus indicated that this DNA-based interaction was targeted to heterochromatin and involved the intact metallointercalator molecule [20]. Additionally, a yeast transcriptomic study using cDNA microarrays identified glutathione, iron and copper related pathways in the cellular response to 56MESS [21]. Specifically, the up-regulated genes were involved in the homeostasis of iron and copper, and the transport of these metal ions across the plasma membrane. This is notable as copper transport has been implicated in the transport of cisplatin [22,23] and may be part of a more generalised response to heavy metals. Down-regulated genes were associated with amino acid metabolism, the yeast stress response, and cellular respiration [21]. Previous studies suggest that other heavy metals affect cell division and when in excess will be stored in the vacuole for use during times of scarcity and as protection from cytotoxicity [24,25]. The varied mechanisms of 56MESS activity suggested by this evidence imply that there are multiple factors at play in a cellular response to 56MESS. In this work, we have used a well-characterized eukaryotic model and a Systems Biology approach that includes both genomic and proteomic analyses to refine our understanding of mechanisms underlying the cellular response to 56MESS relative to cisplatin.

*Saccharomyces cerevisiae* is a single celled eukaryote that is extremely amenable to genetic and other experimental manipulations [26]. As such, *S. cerevisiae* has been used extensively as a model for human cells, with many highly conserved genes, and thus proteins, molecular mechanisms, as well as cellular structures and processes [27]; this has facilitated its use in the screening of potential anticancer agents [28]. In light of the conservation of fundamental mechanisms, the usefulness of this model has been further facilitated by the construction of a commercial yeast deletion mutant library (EUROSCARF deletion library [29]). This library can be used in conjunction with the Affymetrix TAG3 microarray system [28], enabling the identification of gene deletions that alter cellular response to a specific environmental perturbation [26]. The library has previously been used with TAG microarrays, to identify mechanisms potentially involved in the biological response to cisplatin [30]. Here, these arrays enabled assessment of genomic information that could then be more effectively consolidated with proteomic data.

While it is not surprising that 56MESS, a compound designed to interact with DNA is, in part, localised to the nucleus, additional mechanisms also appear to be at work [20,21]. Thus, we hypothesized that the increased efficacy of 56MESS was due to a mode of action potentially quite different to cisplatin. Screening for mutants with increased sensitivity to 56MESS implicated protein trafficking and degradation functions and, as such, the proteome was subsequently examined using high resolution, top-down proteomics (*i.e.*, one- and two-dimensional gel electrophoresis) in direct comparison with cisplatin treated cells. This integrated Systems Biology approach has thus provided insight into the anticancer potential of 56MESS, particularly with regard to its likely alternate mechanisms of cytotoxicity relative to the current therapeutic standard, cisplatin.

## 2. Experimental Section

### 2.1. Reagents and Suppliers

All reagents were of analytical grade or higher, and supplied by Amresco unless otherwise stated. The manufacture of 56MESS was undertaken using published methods [17]. Cisplatin (99.9% purity) was obtained from Sigma (Sydney, Australia).

### 2.2. Media, Yeast Strains and Growth Conditions

Yeast growth was carried out in liquid yeast extract/peptone/dextrose (YEPD) media prepared as described previously [31], with orbital shaking (120 rpm). Stationary incubation of yeast on YEPD agar (2% (w/v)) plates was carried out at 30 °C.

Wildtype BY4743 *S. cerevisiae* (mating type: MATa/MATα, his3Δ1/his3Δ1, leu2Δ0/leu2Δ0, met15Δ0/+, lys2Δ0/+, ura3Δ0/ura3Δ0) was grown to log phase (OD_600_ = 1.0) and exposed to 56MESS to determine the optimal concentration and time span for selecting sensitive mutants. This culture was divided into subcultures and exposed to 56MESS concentrations ranging from 0–8 mM. Aliquots of the exposed cultures were taken every 15 min for the first hour, every 30 min for the second hour, and then hourly for the following 4 h and grown on YEPD plates to establish the loss of cell viability caused by each concentration. The percentage of cell death was measured as the loss in total number of colony forming units present in the culture. 

The homozygous diploid deletion mutant pool in the BY4743 genetic background (Invitrogen, Melbourne, Australia) was used in the TAG microarray experiments [26,32]. Master cultures of the mutant library pool were grown to log phase (OD_600_ = 1.0). Cultures were then exposed to 2 mM 56MESS for 3 h and aliquots from each were tested as described above, to yield a dose response curve [31].

### 2.3. Tag Microarray

Duplicate 50 µL aliquots were taken from cultures treated with 0, 4 and 8 mM 56MESS at 0.25, 0.5, 0.75, 1, 1.5, 2 and 3 h. All aliquots were inoculated into separate 100 mL volumes of fresh YEPD and supplemented daily with fresh YEPD for 3 days (*i.e.*, yielding OD_600_ = 6.0). Cells were then harvested by centrifugation at 600× *g* for 5 min and cell pellets were frozen in liquid nitrogen and stored at −80 °C until DNA extraction was carried out as described previously [33]. 

The UP- and DOWNTAG molecular barcode sequences were amplified separately from 1.5 µg of extracted DNA using previously identified primers (Invitrogen, Melbourne, Australia) [32]. These primers were designed specifically for amplification of the molecular barcodes of the deletion mutants. The PCR protocol used 20 mM tris-HCl (pH 8.4) / 50 mM KCl (PCR buffer from Invitrogen), 0.4 mM of each deoxynucleotide triphosphate, Taq polymerase (20 U mL^−1^), forward and reverse primer (1 µg mL^−1^) and chromosomal DNA (20–30 µg mL^−1^). 

The UP- and DOWNTAG PCR products for each sample were combined and prepared for hybridization as described previously [32]. Briefly, UP- and DOWNTAG PCR products were combined with 1 M NaCl / 66 mM NaH2PO4 / 6.6 mM EDTA (pH 7.4) / 0.005% (w/v) Triton X-100 (6 × SSPE-T) and 400 pmol each of the UP- and DOWNTAG amplified DNA samples. Sample hybridization cocktails were heated at 100 °C for 5 min, cooled on ice for 2 min, injected into an Affymetrix Tag3 GeneChip® high-density oligonucleotide microarray, and hybridized for 16 h at 42 °C with orbital rotation (60 rpm). Washing and staining of chips was carried out as previously described [32] and chips were scanned at 570 nm using an Affymetrix GeneChip® scanner [26,34].

Resultant scanner images of each GeneChip® microarray were quantified for analysis using the 75th percentile of each separate TAG element on the microarray [26]. Mutants were then linked to their corresponding molecular UP- and DOWNTAG marker by GeneChip® Operating Software (Version 1.4) with reference to a GeneFlex® library of TAG sequences.

Raw data were then sorted for perfect match (PM) values. PM values are signal readings associated with the hybridization of each TAG to its exact sequence complement on the microarray. A ratio of solvent signal over treatment signal was calculated; all mutants with a 1.5-fold change (*p* < 0.05) in their complement of UP- and DOWNTAG signals were retained for further testing (Table S1).

### 2.4. Sensitivity Confirmation 

A library of individually stored mutants obtained from the European *S. cerevisiae* Archive for Functional Analysis (EUROSCARF) was used for secondary analysis of the 133 mutants identified by the microarray experiments. Mutants identified as sensitive to 56MESS were each grown in a separate well on a microtitre plate, spiked with glycerol (15% w/v) and stored at −80 °C. Mutants were thawed and replicated onto a fresh YEPD-filled microtitre plate, grown to confluence and incubated an additional 4 h following addition of fresh media. All mutants were then exposed to a solvent control or 2 mM of either 56MESS or cisplatin over 24 h. Well contents were plated onto YEPD agar at times 0, 1, 2, 4, 8 and 24 h. Empty wells were distributed randomly across each mutant plate to indicate potential levels of cross contamination. BY4743 was used as a reference strain with five randomly assigned wells on each microtitre plate. This process was repeated for all mutants. 

### 2.5. Protein Sample Preparation and Extraction

Triplicate BY4743 cultures were grown to OD_600_ = 1.0 and split into three subcultures for treatment with solvent, cisplatin, or 56MESS. Prior to administration of treatments, initial samples were taken from each culture (*i.e.*, 0 h), and then another at 4 h for protein extraction (as well as an aliquot for plating onto YEPD to generate a concurrent kill curve). Samples were centrifuged at 600× *g* for 5 min and the cell pellet was snap frozen in liquid nitrogen before being stored at −80 °C (*i.e.*, AFD; automated frozen disruption). Frozen yeast cell pellets were pulverised to a fine powder using a Mikro-Dismembranator S (Sartorius AG, Goettingen, Germany) before solubilisation (*i.e.*, AFD; automated frozen disruption) [35,36]. All protein fractions were quantified using the EZQ Protein quantification assay (BioRad, Gladesville, Australia) and then stored at −80 °C until needed [37].

### 2.6. 1D SDS Polyacrylamide Gel Electrophoresis

Protein samples were diluted with 2 × sample buffer (0.125 M tris (pH 8.8) / 5% (w/v) SDS / 25% glycerol / 0.0625 M dithiothreitol (DTT) / 0.001% (w/v) bromophenol blue) with a protease inhibitor cocktail (2 µg mL^−1^ each of aprotinin, pepstatin and leupeptin) [35,36]. All samples were diluted to enable uniform total protein loads in every analysis. Gel electrophoresis was carried out as previously described with minor modifications [38,39,40,41]. Once complete, gels were fixed for a minimum of 1 h.

### 2.7. Tris-Tricine Polyacrylamide Gel Electrophoresis

Samples were prepared for tris-tricine PAGE as described above for 1D electrophoresis. Tricine PAGE was carried out as described previously with minor alterations [42,43,44,45]. Resolving gels (17% T, 5.88% C / 1 M tris / 0.1% (w/v) SDS / 13.5% (w/v) glycerol / 0.033% (v/v) TEMED / 0.033% (w/v) APS) were cast and allowed to polymerize overnight. A stacking gel prepared as described above was then overlaid on the resolving gel and formed into wells. Anodic buffer consisted of 0.2 M tris (pH 8.9) and the cathodic buffer of 0.1 M tris, 0.1 M tricine and 0.1% (w/v) SDS. Gel electrophoresis was carried out at 4 °C for 4 h at a constant 50 mA and a maximum of 120 V. Gels were then fixed overnight (min 18 h). 

### 2.8. 2D SDS Polyacrylamide Gel Electrophoresis

2D SDS PAGE (2DE) was carried out as described previously [35,36,41,44,45,46,47,48]. Briefly, following AFD, the resulting ‘powdered’ cell pellets were solubilised in 8 M urea, 2 M thiourea and 4% (w/v) 3-[(3-cholamidopropyl)dimethylammonio]-2-hydroxy-1-propanesulfonate (CHAPS) with a protease inhibitor cocktail (2 µg mL^−1^ each of aprotinin, pepstatin and leupeptin) [35,36]. Each sample was reduced and alkylated as described previously and a volume equivalent to 100 µg of protein/sample was passively hydrated into 7 cm 3–10 non-linear immobilised pH gradient (IPG) strips (Bio-Rad) for 16 h at room temperature (RT) [35,36,41,44,45,46,47,48].

Isoelectric focusing (IEF) of the hydrated IPG strips was carried out as described previously at 17 °C with an initial desalting step at 250 V for 15 min [35,36,41,44,45,46,47,48]. Voltage was linearly ramped for 2 h to 4000 V with wick changes as required to maintain ramping (*i.e.*, five in this instance). IPG strips were then focused at 4000 V for 37,500 Vh and stored at −30 °C until required. Focused strips were equilibrated first in 2% DTT, then 2.5% (w/v) acrylamide and thoroughly drained of buffer [35,36,41,44,45,46,47,48]. Proteins in the IPG strips were then resolved using stacking and resolving gels prepared as described above, and overlaid with agarose (375 mM tris buffer (pH 8.8) / 0.5% (w/v) low melting agarose / 0.1% (w/v) SDS / 0.01% (v/v) bromophenol blue). Electrophoresis and fixation were carried out as described for 1D SDS PAGE. 

### 2.9. Staining and Quantitative Analysis

Following fixation, gels were washed in distilled water for 3 × 20 min, stained overnight with SYPRO Ruby (SR) and washed with fixative for 3 × 20 min. Gels were then washed for 3 × 20 min in distilled water and imaged using a FLA-9000 fluorescence imager (Ex: 473 nm; Em: 510 nm) (Fujifilm). Fluorescence imaging and quantitative image analysis were carried out as described previously [35,36,41,44,46,47,48]; Delta 2D (Decodon, Greifswald, Germany) was used to analyse the 2D gel images.

### 2.10. Mass Spectrometric Analysis

Proteins differing substantially in abundance, as identified by Delta 2D (Decodon), were assessed as to their experimental isoelectric points and molecular weights based on the parallel use of calibration standards during 2DE (2-D SDS-PAGE Standards (Bio-Rad, Gladesville, Australia) and PageRuler Unstained Protein Ladder (Thermo Scientific, Scoresby, Australia)). Protein spots that were uniquely present or absent (*i.e.*, above or below the minimal level of detection, respectively) in one experimental condition over the others (by definition, ‘all or none’ changes) were selected for mass spectrometric (MS) analysis and identification. Protein spots were excised and prepared for tryptic digest and peptide extraction as described previously [41] with minor alterations [45,49]. Following peptide extraction, 15 µL was transferred to an autosampler vial for LC/MS/MS analysis. The MS/MS data files produced by the QSTAR were queried using Mascot Daemon (version 2.4, provided by the Walter and Elisa Hall Institute [50]) and searched against the LudwigNR database (comprised of the UniProt, plasmoDB and Ensembl databases (vQ111. 16,818,973 sequences; 5,891,363,821 residues) with the parameter settings described previously [41] and the additional variable modifications: Carbamidomethyl. The results of the search were then filtered by including only protein hits with at least one unique peptide and excluding peptide hits with a *p*-value greater than 0.05. Peptides identified by Mascot were further validated by manual inspection of the MS/MS spectra for the peptide to ensure the b- and y-ion series were sufficiently extensive for an accurate identification. 

### 2.11. Bioinformatics

Genes identified and confirmed as contributing to 56MESS sensitivity when deleted were analysed for function, localization and molecular interactions using the Yeast Proteome Database (YPD) [51] and Saccharomyces Genome Database (SGB) [52]. The molecular weight and isoelectric points of the products of these genes were also sourced. Based on these classifications and descriptions, genes were categorised according to common function. The SGD Go Slim mapping tool was also used to categorise genes from this and a previously published study [21] for comparison.

## 3. Results

**Figure 1:**
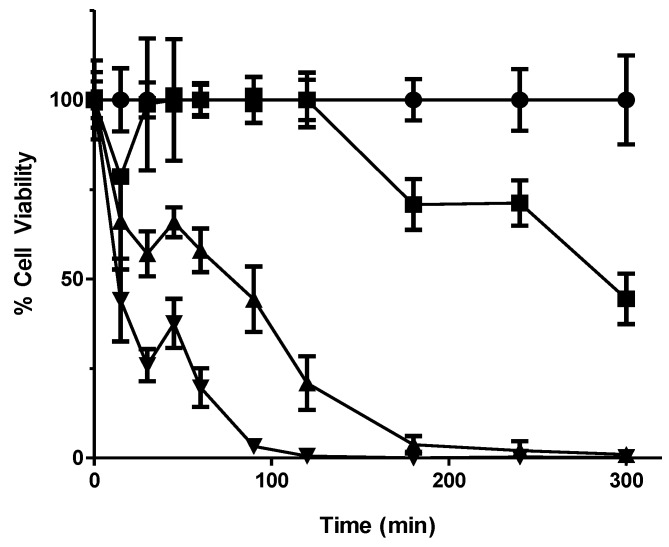
Kill curve using wildtype BY4743 to determine yeast deletion mutant pool test parameters. A culture was grown to OD 1, divided into separate subcultures and exposed to 0, 4, 6 and 8 mM of 56MESS. Treated culture samples were plated onto yeast extract/peptone/dextrose (YEPD) plates over 5 h. The resultant colonies after 48 h of incubation at 30 °C were counted to determine the cell viability for each culture exposed to each 56MESS concentration. Error bars represent standard error of the mean (SEM) (n = 3) ● Solvent; ■ 4 mM 56MESS; ▲ 6 mM 56MESS; ▼ 8 mM 56MESS.

**Figure 2:**
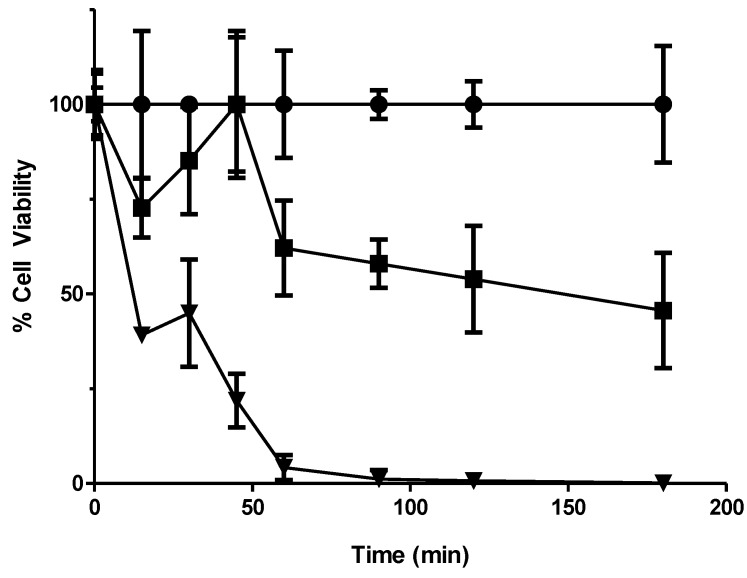
Kill curve using the yeast deletion mutant pool conducted to relate sampling times to losses in cell viability. Cultures were exposed to 0, 4 and 8 mM 56MESS and aliquots taken over 3 h were plated onto YEPD. Following 48 h of incubation at 30 °C, plated colonies were counted to determine cell viability. Error bars represent SEM (n = 3). ● Solvent; ■ 4 mM 56MESS; ▼ 8 mM 56MESS.

Drug treatment concentrations were determined by kill curve analysis of wild type yeast and the deletion library was then exposed to these concentrations (Figure 1 and Figure 2). The DNA containing the molecular TAG3 labels of the sensitive mutants was then extracted from culture, amplified by PCR and hybridised to a TAG3 microarray. This data were used to produce a list of potentially sensitive deletion mutants having a statistically significant ≥1.5-fold change in signal between control and treated samples. These genes were categorised according to predicted function and localisation of the proteins they encode (Figure S1). Following verification of sensitivity via microtitre plate assay and individual exposure to 56MESS, a subtotal of 48 gene deletion mutants was confirmed as 56MESS-sensitive. 

**Figure 3:**
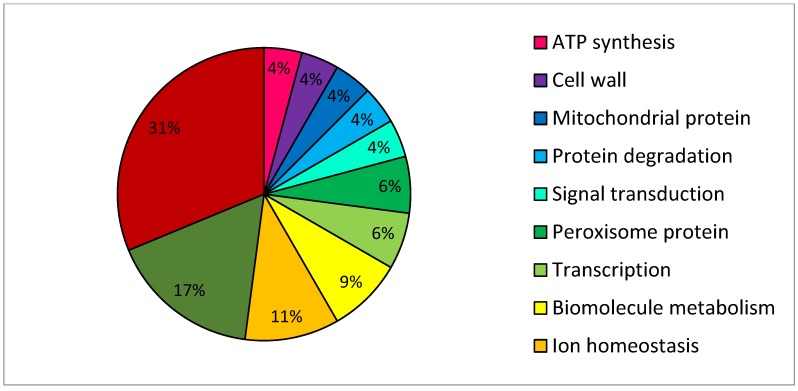
Distribution of gene function amongst confirmed 56MESS sensitive deletion mutants. The top 200 sensitive mutants as identified by microarray were submitted to individual 56MESS sensitivity testing in microtitre plates. A subset of 48 mutants were confirmed for sensitivity to 56MESS.

The functional group with the highest representation (31.3%) among the confirmed sensitive mutants was the vacuolar trafficking system (Figure 3), representing the primary cyclic exo- and endocytotic pathways within these cells. Other functional groups represented in the 56MESS sensitive deletion mutant collection included cell cycle genes (16.7%) and those involved in ion homeostasis (10.4%). Among the other groups, genes linked to biomolecule metabolism (8.3%), the peroxisome (6.3%) and protein degradation (4.2%) also increased sensitivity to 56MESS when deleted. No major DNA repair mechanisms were present among the confirmed sensitive gene deletions in stark contrast to what is known about the mechanism of action of cisplatin. Taking all genes involved in protein trafficking, handling and/or degradation into account, there is a high proportion (41.8% total) that contribute to 56MESS sensitivity. This potentially implicates these processes and proteins themselves as significant potential targets of 56MESS. This also indicates that DNA may not be the sole target of the anticancer compound 56MESS. Furthermore, as proteins (*i.e.*, gene products) are themselves the active arbiters of cellular function and response, it was also deemed necessary to analyse the proteome using both 1- and 2D SDS PAGE techniques. As cisplatin is considered the leading industry standard, it was also included for direct comparison with 56MESS.

**Figure 4:**
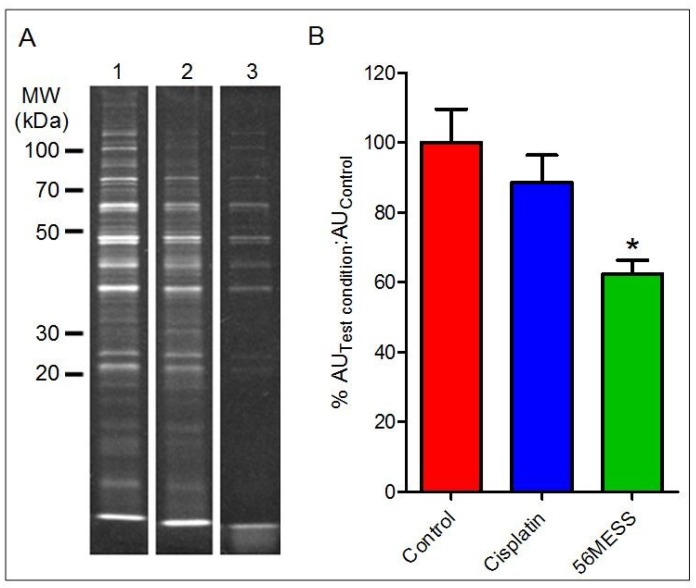
1D SDS-PAGE of total protein extracts after 4 h exposure of cells to control, cisplatin or 56MESS treatments. Equal protein loads as measured by EZQ were resolved and detected with SYPRO Ruby and gel images were analysed using Multi Gauge. (**A**) Lane 1: Control; Lane 2: Cisplatin; Lane 3: 56MESS; (**B**) Ratio of the fluorescent signal of the test condition protein sample over the fluorescent signal of the control protein sample after 4 h of drug exposure. 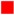 control; 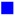 1 mM cisplatin; 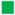 2 mM 56MESS. Error bars represent SEM. ‘*’ indicates *p* = 0.0064 (n = 3).

Yeast proteins from cultures exposed to solvent (*i.e.*, control), cisplatin, and 56MESS for 4 h were resolved using 1D SDS-PAGE (Figure 4A). Concentrations were selected based on cell viability in response to a range of 56MESS and cisplatin concentrations, and also produced the most similar decreases in viability (Figure S2). The cisplatin-exposed samples yielded a protein band pattern comparable to the control after 4 h (Figure 4A). The addition of cisplatin to growing conditions did not alter the protein complement of yeast in comparison to the control; the addition of 56MESS reduced the number of detectable proteins. When this was quantified as a ratio of fluorescent signal associated with samples treated either with cisplatin or 56MESS over that of the total protein extracted from control samples, it indicated that significantly less protein was detected in the 56MESS exposed samples (*p* = 0.0064). Furthermore, it was also evident that while there was less protein in the 25–100 kDa range, the samples that had been exposed to 56MESS also had a diffuse protein band located beyond the dye front. Quantification of this low molecular weight material indicated that a high proportion (40 ± 1% of total signal) of the total protein sample was present in the diffuse band, thus accounting for the differences in the density of resolved proteins despite equivalent total protein loads of each sample (*i.e.*, as determined using a general protein detection reagent) (Figure 4B). No similar band was detected in the cisplatin-exposed sample.

**Figure 5:**
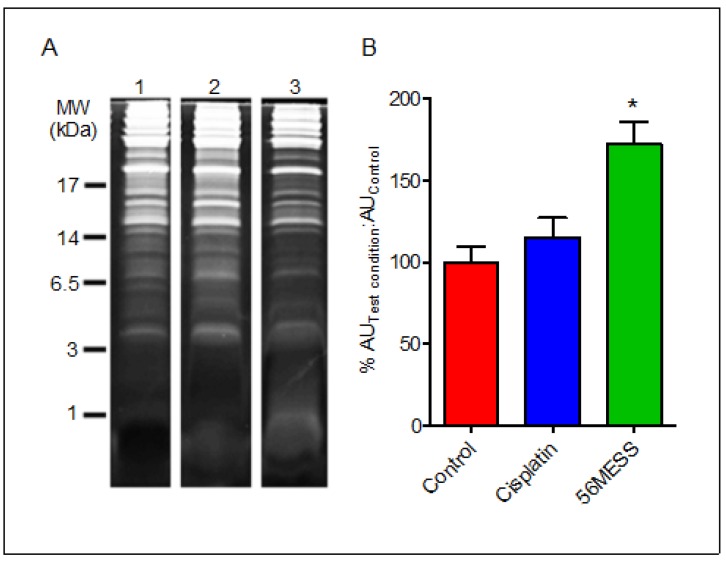
(**A**) Tris-tricine PAGE gel of proteins extracted from solvent, cisplatin and 56MESS exposed cultures. Equal protein loads as measured by EZQ were resolved and detected with SYPRO Ruby and gel images were analysed using Multi Gauge. 1. Control; 2. Cisplatin; 3. 56MESS; (**B**) Ratio of the fluorescent signal of the test condition protein sample to that of the control protein sample after 4 h of drug exposure. Quantitation was limited to the 1 kDa region of the lane to focus on the fluorescence associated with the low molecular weight material in the 56MESS sample. 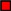 Control; 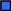 1 mM cisplatin; 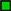 2 mM 56MESS. Error bars represent SEM. ‘*’ indicates *p* < 0.05 (n = 3).

The presence of low molecular weight (MW) fragments suggested that a method capable of resolving protein/peptides in this MW range was required. Thus, tris-tricine electrophoresis was employed to resolve any low MW peptide material within these samples. The diffuse band below the dye front in the 56MESS 1D gel lane (Figure 5A) was also apparent in the 1 kDa range following resolution using tris-tricine gels, consistent with peptide-size protein fragments and/or the end products of protein degradation (Figure 5). Furthermore, extracts from the control and cisplatin exposed samples did not have any such prominent low molecular weight band. Quantification of this diffuse protein band indicated that this proteinaceous material was ~1.8-fold more abundant than in the corresponding solvent control sample extracts as indicated by fluorescent signal intensity (Figure 5B).

**Figure 6:**
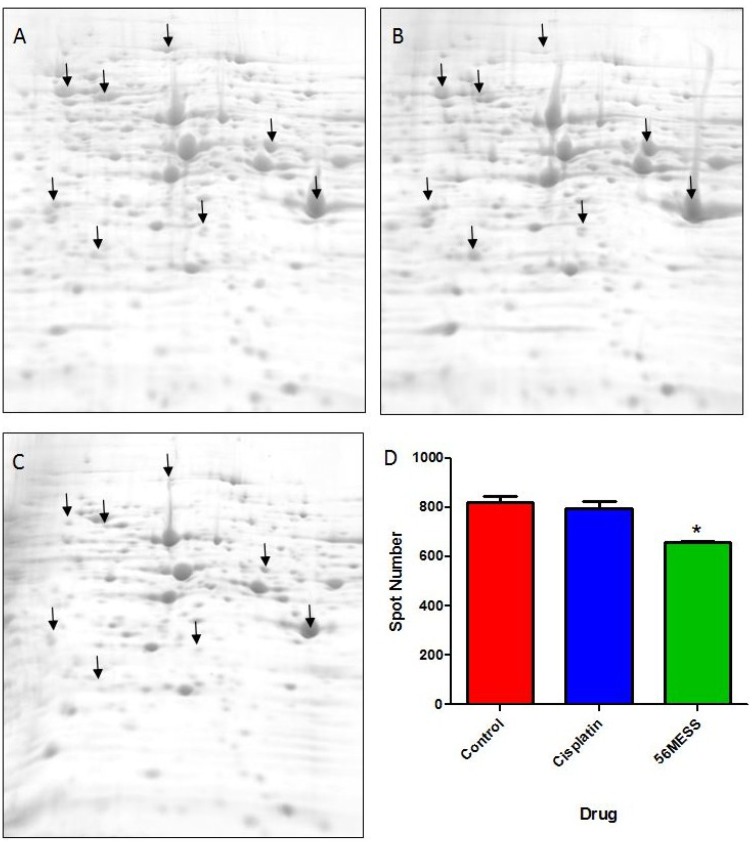
Representative 2DE gel images for each of the proteomes extracted from a control yeast culture (**A**) and cultures exposed to cisplatin (**B**) or 56MESS (**C**), and protein spot counts (**D**) Error bars represent SEM. ‘*’ indicates *p* = 0.0041 (n = 3). Arrows indicate some representative protein spots (*i.e.*, protein species) that are differentially abundant between experimental and control conditions.

As substantial changes in protein abundance (e.g., increased protein degradation) were suggested by the initial proteomic assessments (Figure 4 and Figure 5), a more comprehensive examination of the yeast proteome following exposure to 56MESS was undertaken using a highly refined, quantitative top-down analytical approach [35,41,44,45,46] — 2D gel electrophoresis (2DE) — coupled with mass spectrometry for protein identification (2DE-MS) (Figure 6). The resulting gel images were analysed to determine the total number of protein spots (*i.e.*, species) resolved and any differential changes in spot volume (*i.e.*, amount of protein) between conditions. Extracts from cultures exposed to 56MESS had the lowest number of detectable proteins (658 ± 6, mean ± SEM), and this was significantly different (*p* = 0.0041) from the cisplatin-treated and control cultures (820 ± 26 and 797 ± 27, respectively; Figure 6D). This represents a potential change in expression, processing, modification or degradation of ~20% of the proteome. This is far from a minor effect. Furthermore, direct comparison of individual spots between treatments showed that the majority were of lower volume in the 56MESS samples than in the extracts from cisplatin-treated cells. Differentially abundant protein spots (Figure 6; indicated with arrows) were further characterised according to apparent molecular weight, isoelectric point, and fold-change following drug treatment (Tables S2 and S3). Select protein spots were submitted for MS analysis based on all or none changes between the control and 56MESS conditions. Several proteins that were more abundant in the 56MESS samples relative to the control were identified as heat shock proteins (Table 1). Two enolase proteins and a pyruvate kinase involved in glycolysis and gluconeogenesis, as well as a ribosomal protein involved in translational elongation were also identified (Table 1). There were no overlapping hits between the 48 confirmed 56MESS sensitive mutants (Figure 3; Table S4) and the proteins identified by 2DE-MS. However, the experimental molecular weight and isoelectric point values of differentially abundant protein spots can be compared with the reported values of the sensitive deletion mutants and some showed good correlation (marked with a “*” in Table S4). 

**Table 1:** Differentially expressed proteins submitted for MS analysis. All identified proteins were *S. cerevisiae* in origin.

Mascot ID	Gene	Score	Theoretical		Observed		Coverage	Peptides	E-value
P10591	SSA1/member of the heat shock family	1551	69.6	5.0	72.2	4.4	42%	K.ATAGDTHLGGEDFDNR.L	9.50E-06
								K.SQVDEIVLVGGSTR.I	6.20E-05
								K.LVTDYFNGKEPNR.S + Deamidated (NQ)	3.80E-04
								K.NQAAMNPSNTVFDAK.R + Oxidation (M)	1.70E-03
								R.SINPDEAVAYGAAVQAAILTGDESSK.T	2.00E-03
								K.DAGTIAGLNVLR.I	2.30E-03
								K.ATAGDTHLGGEDFDNR.L	1.00E-02
								K.ELQDIANPIMSK.L + Oxidation (M)	1.00E-02
								R.IINEPTAAAIAYGLDK.K	4.90E-02
P00925	ENO2/Enolase II, phosphopyruvate hydratase	997	47	5.6	31.4	5.1	37%	K.TAGIQIVADDLTVTNPAR.I	1.10E-05
								K.DGKYDLDFKNPESDK.S	2.80E-05
								K.AVDDFLLSLDGTANK.S	4.40E-05
								R.SGETEDTFIADLVVG.L	5.80E-05
								K.LGANAILGVSMAAAR.A + Oxidation (M)	8.40E-04
								K.IGLDCASSEFFK.D + Propionamide (C)	2.30E-03
								K.VNQIGTLSESIK.A	3.30E-02
P00560	PGK1/3-phosphoglycerate kinase	494	44.7	7.1	19.7	5.8	23%	K.ASAPGSVILLENLR.Y	1.70E-02
								K.VLENTEIGDSIFDK.A	1.30E-05
E7NIQ9	ENO1/Enolase I, phosphopyruvate hydratase	309	34	5.7	17.6	6.7	16%	R.GNPTVEVELTTEK.G	1.60E-02
								K.AVDDFLLSLDGTANK.S	2.60E-04
P10592	SSA2/ stress induced heat shock protein	4809	69.4	5.0	72.2	4.8	67%	K.KAEETIAWLDSNTTATKEEFDDQLK.E	1.50E-08
								K.NTISEAGDKLEQADKDAVTK.K + Deamidated (NQ)	5.90E-08
								R.SINPDEAVAYGAAVQAAILTGDESSK.T	2.40E-07
								K.LDKSQVDEIVLVGGSTR.I	3.40E-07
								K.ATAGDTHLGGEDFDNR.L	1.80E-06
								K.AVGIDLGTTYSCVAHFSNDR.V + Propionamide (C)	3.20E-06
								K.NQAAMNPANTVFDAKR.L + Oxidation (M)	4.90E-06
								K.TQDLLLLDVAPLSLGIETAGGVMTK.L + Oxidation (M)	7.70E-06
								R.IINEPTAAAIAYGLDKK.G	7.80E-06
								K.LVTDYFNGKEPNR.S + Deamidated (NQ)	3.40E-05
								K.KSEVFSTYADNQPGVLIQVFEGER.A	8.90E-05
								K.ATAGDTHLGGEDFDNR.L	1.10E-04
								K.MKETAESYLGAK.V + Oxidation (M)	2.40E-04
								K.SQVDEIVLVGGSTR.I	3.00E-04
								K.NFTPEQISSMVLGK.M + Oxidation (M)	3.60E-04
								K.KAEETIAWLDSNTTATKEEFDDQLK.E	1.50E-08
P32589	SSE1/ATPase involved in the Hsp90 complex	1986	77.3	5.2	82.3	4.9	36%	F.GLDLGNNNSVLAVAR.N	6.40E-07
								K.KDDLTIVAHTFGLDAK.K	1.00E-06
								K.DDLTIVAHTFGLDAK.K	6.90E-06
								K.PLSTTLNQDEAIAK.G	1.00E-05
								F.GLDLGNNNSVLAVAR.N + Deamidated (NQ)	2.70E-05
								K.HVFSATQLAAMFIDK.V + Oxidation (M)	1.70E-04
								R.EELEELVKPLLER.V	2.70E-04
								R.GIDIVVNEVSNR.S	4.90E-04
								K.QVEDEDHMEVFPAGSSFPSTK.L + Oxidation (M)	5.20E-04
								R.IVNDVTAAGVSYGIFK.T	7.50E-04
								R.KNTLEEYIYTLR.G	8.00E-04
								K.QSISEAFGKPLSTTLNQDEAIAK.G	1.30E-03
								R.GKLEEEYAPFASDAEK.T	2.40E-03
								K.YEELASLGNIIR.G	2.60E-03
								R.IIGLDYHHPDFEQESK.H	3.60E-03
								K.GAAFICAIHSPTLR.V + Propionamide (C)	3.90E-03
								K.LNELIEKENEMLAQDK.L + Oxidation (M)	2.40E-02
E7KFS3	HSP60/Chaperonin mediates protein refolding after stress	2189	60.7	5.2	61.2	4.8	52%	K.TNEAAGDGTTSATVLGR.A	5.30E-10
								K.QIIENAGEEGSVIIGK.L	3.90E-08
								K.EITTSEEIAQVATISANGDSHVGK.L + Deamidated (NQ)	1.20E-06
								K.GVETLAEAVAATLGPK.G	2.80E-06
								K.SEYTDMLATGIIDPFK.V + Oxidation (M)	1.50E-05
								R.TLEDELEVTEGMR.F + Oxidation (M)	1.00E-04
								K.VEFEKPLLLLSEK.K	1.20E-04
								K.DRYDDALNATR.A	1.60E-04
								R.VGGASEVEVGEK.K	5.00E-04
								K.GSIDITTTNSYEK.E + Deamidated (NQ)	2.10E-03
								R.VLDEVVVDNFDQK.L	2.00E-02
P05317	RPP0/Conserved ribosomal protein involved in translational elongation	582	33.7	4.8	24.4	4.7	22%	K.SLFVVGVDNVSSQQMHEVR.K	3.70E-09
								K.TSFFQALGVPTK.I	8.50E-04
								K.GNVGFVFTNEPLTEIK.N	3.30E-03
								R.GTIEIVSDVK.V	4.20E-02
P00549	CDC19/Pyruvate kinase	3074	54.5	7.6	57.0	7.2	74%	F.VFEKEPVSDWTDDVEAR.I	8.50E-04
								K.ACDDKIMYVDYK.N + Oxidation (M); Propionamide (C)	2.90E-02
								K.AIIVLSTSGTTPR.L	4.30E-04
								K.EPVSDWTDDVEAR.I	3.60E-04
								K.GVNLPGTDVDLPALSEK.D	1.20E-02
								K.IENQQGVNNFDEILK.V	3.00E-03
								K.KGDTYVSIQGFK.A	3.60E-07
								K.NGVHMVFASFIR.T + Deamidated (NQ); Oxidation (M)	4.00E-02
								K.PTSTTETVAASAVAAVFEQK.A	4.10E-04
								K.SEELYPGRPLAIALDTK.G	2.60E-02
								K.SNLAGKPVICATQMLESMTYNPR.P + 2 Oxidation (M); Propionamide (C)	2.10E-05
								K.TNNPETLVALR.K	2.10E-03
								R.AEVSDVGNAILDGADCVMLSGETAK.G + Oxidation (M); Propionamide (C)	3.20E-04
								R.EVLGEQGKDVK.I	4.50E-02
								R.KSEELYPGRPLAIALDTK.G	2.70E-04
								R.LTSLNVVAGSDLR.R	4.40E-04
								R.NCTPKPTSTTETVAASAVAAVFEQK.A + Propionamide (C)	5.50E-03

## 4. Discussion

Proteome analysis of yeast challenged with 56MESS identified a protein-based response that was demonstrably different from the cellular response to the current clinical treatment standard, cisplatin. Total numbers of protein species as well as overall protein abundance were reduced, suggesting a role for 56MESS in triggering protein degradation or affecting protein translation. Of the differentially abundant protein species, most were primarily implicated in the known generalised stress response.

### 4.1. Yeast as a Tool for Systems Biology Analyses of Drug Actions

As a simple and readily available eukaryote, *S. cerevisiae* is used to investigate fundamental processes of wider biological relevance [53]. High rates of homologous recombination and a completely sequenced genome facilitate high levels of experimental control over genetic content. This is particularly relevant as 31% of yeast proteins have a human orthologue and 50% of human disease genes have yeast orthologues [54]. Yeast is thus a valuable model to initially identify the potential targets and/or mechanisms of action of novel anticancer agents.

As cancer poses a continuing threat to the population, innovative chemotherapeutic approaches must be sought [1,55]. The novel anticancer compound, 56MESS, has a potent activity in both cisplatin-susceptible and -resistant cell lines. However, as unknown mechanisms of action are often limiting factors in the progression of compounds to approved treatments, this compound requires further characterisation.

### 4.2. The Yeast Response to 56MESS

An initial broader spectrum, high throughput microarray approach was useful as a preliminary investigation into the activity of 56MESS. The deletion library was a valuable resource for the concurrent interrogation of the entire deletion mutant complement of the yeast genome. Each of these gene deletion mutants is identified by a molecular barcode, enabling isolation and identification of strains (and thus genes) potentially involved in the response to different physiological challenges [26]. Screening the yeast deletion mutant library, which represented 96% of the ORFs, reduced the field of investigation from approximately 6000 genes in the complete genome [56,57] to 133 mutants (Figure S1) and facilitated the individual examination of mutant sensitivities. This resulted in 48 confirmed sensitive deletion mutants (Figure 3; Table S4).

Deletion mutant studies of cisplatin primarily have identified genes involved in different repair pathways (*i.e.*, nucleotide excision, recombinational, post replication, DNA inter-strand cross-link, and oxidative damage); these gene deletions sensitize yeast to cisplatin, suggesting DNA as an important target and its repair as vital for cell survival [30]. None of these genes or processes were implicated by the 56MESS sensitive deletion mutants. The overlap between genes affecting cisplatin sensitivity and those influencing 56MESS sensitivity was marginal at 4%. Only two genes were common in the 56MESS and cisplatin sensitive deletion mutants, IMP2` and HAL5 [30]. IMP2` is a transcriptional activator involved in protection against oxidative DNA damage as well as ion homeostasis [58]. HAL5 is a protein kinase involved in transport regulation, which causes sensitivity to acidic pH and cations when deleted [59]. Both genes have regulatory functions and could represent a general cell signalling response to the cationic charge of both drugs rather than to the cytotoxic effects of either. Thus, identification of common genes related to the cellular responses to cisplatin and 56MESS does not preclude different mechanisms of action for these compounds.

Here, deletion of various vacuolar protein sorting (VPS) genes resulted in increased sensitivity to 56MESS (Figure 3, Table S1). Traffic from the Golgi to the vacuole relies on products of VPS genes [60], and these are also required for broad resistance to oxidative stress [61]. VAM7, VTC1, VPS30 and VPS41 genes (Figure 3; Table S4) code for subunits of vacuolar transporter chaperones, receptors, tethering complexes and a protein involved in intra-vacuolar processing [62,63,64,65,66,67]. Other related gene deletions also implicated wider protein sorting and trafficking pathways in 56MESS sensitivity including membrane fusion, ubiquitin dependent protein sorting and Golgi protein recycling [68,69]. In direct contrast to 56MESS, the deletion of vesicle-mediated transport/trafficking related genes increased resistance to cisplatin when deleted [70]. Cumulatively, these results suggest that intracellular protein trafficking mechanisms are particularly critical for resistance to 56MESS such that inhibition of these functions results in sensitivity to this agent (Figure 3; Table S4). The prevalence of protein handling genes as TAG3 microarray hits suggested that analysis of the yeast proteome following 56MESS challenge would be useful.

Exposure to 56MESS decreased the number of detectable protein species (Figure 4, Figure 5 and Figure 6). The exact mechanism of this broad protein depletion is unclear. However, as the overall abundance of proteins was affected, it is possible that 56MESS is interacting with cellular components other than, or in addition to, DNA [17,71]. Considering the low molecular weight protein fragments detected (Figure 4), it is plausible that 56MESS exposure induced some type of protein degradation and/or transcriptional stall. Thus, protein trafficking, degradation, or transcriptional blockade appear to be potentially critical mechanisms in the response to 56MESS; whether this is in response to the compound specifically or damage caused to cellular components by 56MESS, remains to be determined. Thus, rather than DNA damage, more direct effects of 56MESS on the proteome may be the critical factor in the loss of cell viability.

The identification of different heat shock proteins, that both increase and decrease in abundance, neither confirms nor eliminates protein as a target of 56MESS, but are indicative of cellular stress in response to the compound (Table 1). Furthermore, increased amounts of ENO and PGK proteins that are associated with glycolysis and gluconeogenesis [72], implicate glucose metabolism in the anticancer effects of 56MESS. Mammalian tumours demonstrate increased rates of glycolysis for ATP production in a hypoxic environment [73,74]. By altering the abundance of glycolytic proteins, 56MESS may affect the ability of tumour cells to generate energy thus limiting further growth. Decreased amounts of the translational elongation protein, RRP0p, are, however, consistent with the working hypothesis that the inhibition of transcription has a role in the yeast response to 56MESS. There is little to no correlation between the proteins identified by 2DE-MS and those identified using the deletion mutant library. However, some experimentally determined molecular weight and pI values of differentially abundant proteins (Tables S2 and S3) are comparable to potential gene products (*i.e.*, proteins) identified using the deletion mutant library (Table S4). Thus, were somewhat less stringent criteria used for 2DE ‘spot’ selection (*i.e.*, for subsequent MS analyses), genes/proteins common to both the genomic and proteomic datasets may have been identified. However, using the low stringency of microarray analyses (*i.e.*, identifying all changes ≥1.5-fold) would require the MS analysis of 76% of the detectable proteome. Obviously this would not have been an efficient means to define mechanisms of action. The routine initial analysis used here indicated involvement of proteins including enolase, phosphopyruvate hydratases, and heat shock proteins that should be further assessed in larger scale future analyses. 

The gene products identified here using genomic and proteomic approaches can also be compared to transcriptomic data from yeast exposed to 56MESS [21]. There are no common genes/proteins between that previous study and the results here; however, Yeast GO-Sim Mapping indicated a number of overlapping cellular processes. Ion transport represented 8.3% of the genes identified in this study compared to 15.4% in the transcriptomic data; this was likely implicated as cells attempt to sequester 56MESS in organelles for degradation to counter its toxicity. This is mirrored by cellular ion homeostasis: 8.3 and 12.1%, respectively. The gene ontology term, ‘response to chemical’ represented by 16.7% (genomic) and 7.7% (transcriptomic) of genes in each study, is also common, as is lipid metabolic process (8.3 and 5.5%, respectively) and organelle fission (4.2 and 3.3%, respectively). Nucleobase small molecule metabolism (6.3% / 3.3%) and transcription from RNA polymerase II (12.5% / 2.2%) were also mapped to both transcriptomic and proteomic datasets. Transmembrane transport, however, was found in each of the transcriptomic, genomic and proteomic datasets at 9.9%, 6.3% and 33.3%, respectively. Carbohydrate metabolic process and mitochondrial organisation were also common to all three datasets although this was represented by only a single gene in the genomic and transcriptomic lists, respectively. Many of these common processes could be related to gathering resources in response to chemical insult. The processes common to both genomic and proteomic datasets included protein folding, protein targeting, vacuole organisation and organelle fusion. Many of the differences between the transcriptomic study [21] and the results here could arise from the difference between challenging a fully functioning cell and deleting a gene entirely before challenge. That there were very few common genes between the genomic and transcriptomic studies is likely due to the dissimilar targets of these techniques; while microarrays assess changes in mRNA expression triggered by an external stimulus, gene deletion removes a protein and alters one or more pathways (*i.e*., likely broader responses in terms of a system already in stress). The changes in cell viability and protein abundance as measured by proteomics may thus be a more rational representation of the yeast response to 56MESS as they correspond directly to a struggle to maintain cellular functions using an unaltered genome. The effect of post-translational modifications must also not be ignored. As transcriptomic techniques can only detect relative changes in gene expression, gene products that are transcribed but remain inactive pending post-translational modification would also never be detected by these methods. This suggests that the real strength of genomic, transcriptomic and proteomic methods is their use collectively, in understanding changes across the system in order to focus in on and identify those specifically critical to the physiological challenge in question. It may also be that specific mechanisms tend to be obscured by the use of genomic or transcriptomic analyses as these assess molecules that tend to (i) be distinct from active cellular functions; (ii) not to correlate directly with protein levels or active species (e.g., post-translationally modified); and (iii) undergo transient changes in expression as systems seek a new equilibrium in response to physiological challenges. 

While proteomic studies to characterise chemotherapeutic triggered changes in protein abundance primarily focus on mammalian cell lines [75], the interactions of individual proteins with cisplatin adducted DNA in yeast have been highlighted [23,76,77,78,79]. The proteins are usually critical for DNA repair and have a high affinity for cisplatin adducted DNA [76,80,81]. Some cisplatin sensitivity is mediated by Ctr1p, a high affinity copper transporter [23]; loss of this protein results in increased yeast resistance to cisplatin, possibly as a result of reduced influx and accumulation of cisplatin [23]. Proteasome inhibition also increases cisplatin resistance [82]. Screening for cisplatin-sensitive mutants using a non-essential haploid mutant library also implicated the ubiquitin-proteasome pathway [83]. While this suggested a link between DNA repair and degradation processes in the yeast response to cisplatin, an effect on the resolved proteome was not apparent (Figure 6). 

Overall, in terms of mechanisms of action, the results concerning 56MESS are informative in comparison to cisplatin, but also relative to other intercalators developed as anticancer therapeutics. Intercalator-protein interactions have been described before, and include effects on double strand breaks, inhibition of ribosomal RNA formation, metabolic processes of proteins and nucleic acids, cytoskeleton organisation, and the ubiquitin proteasome pathway [84,85,86,87,88]. Thus, intercalator–protein interactions are likely, usually with a specific or subsequent association with DNA. The effects of 56MESS identified here, suggest a role for such alternate intercalators in the lifecycle of proteins, perhaps at or near synthesis. Thus, the current results support the idea that intercalators interact directly with proteins, and that 56MESS affects proteins in a way that is different to both cisplatin and other intercalators. It is also possible that some intercalators specifically affect transcription of particular gene products; binding specificity can be via interaction with guanine bases in the minor groove or targeting of GC-rich regions [89,90,91]. However, targeting a particular sequence of DNA is far removed from targeting an actual gene sequence. Longer sequences are required to focus the interaction of intercalators to a specific site [92,93]. While intercalation is not yet selective for specific sites on DNA and thus for specific genes (and, subsequently, gene products), there is potential for this specificity to be developed. 56MESS may thus function in a similar albeit less selective manner, affecting a much wider range of genes and subsequent gene products.

## 5. Conclusions

The yeast model has proven informative in terms of exploring potential mechanisms of drug action using an integrated genomic and chemical proteomic approach, and in assessing the similarities and differences in such ‘Omic’ datasets. The differences in mechanism of action of 56MESS relative to cisplatin are thus now characterized according to multiple criteria; rather than DNA repair, gene deletions in protein trafficking pathways induced sensitivity to 56MESS. Higher drug concentrations were used to induce this sensitivity than comparable studies; however, the yeast cell wall and potentially poor metal uptake are possible causes for this difference. A top-down proteomic analysis yielded evidence of protein degradation or interruption of synthesis. Thus, the mechanisms underlying the superior cytotoxicity of 56MESS are very different from those of the widely used clinical standard, cisplatin. This suggests that use of 56MESS in chemotherapy could potentially counter cisplatin resistance in many cancers. The wider implication of these integrated molecular assessments is that 56MESS may provide an avenue for more effective treatment, and for further development of targeted therapeutics. Although additional analyses using mammalian cells are clearly required, the yeast work has now substantially focused this research on alternative targets for next generation anticancer agents. In this regard, the results also emphasize the importance of validating findings at the protein level, indicating that top-down proteomics should constitute a more routine and expected role in studies utilising high-throughput genomic and transcriptomic analyses. 

## Supplementary Materials

Supplementary File 1

## Abbreviations

56MESS: [(5,6-dimethyl-1,10-phenanthroline)(1*S*,2*S*-diaminocyclohexane)platinum(II)]^2+^
AFD: automated frozen disruption
Camp: cyclic adenosine monophosphate
CHAPS: 3-[(3-cholamidopropyl)dimethylammonio]-2-hydroxy-1-propanesulfonate
DTT: dithiothreitol
IEF: isoelectric focusing
IPG: immobilised pH gradient
MW: molecular weight
PM: perfect match
RT: room temperature
SDS PAGE: sodium dodecyl sulphate polyacrylamide gel electrophoresis
SGD: *Saccharomyces* Genome Database
SNARE: Soluble *N*-ethylmaleimide sensitive fusion protein Attachment Protein Receptor
SRXRF: synchrotron radiation X-ray fluorescence
YPD: Yeast Proteome Database
