# Supplementary material for: A Systems Biology Approach to Understanding the Mechanisms of Action of an Alternative Anticancer Compound in Comparison to Cisplatin
Source: Proteomes. 2014 Nov 10;2(4):501–26. doi: 10.3390/proteomes2040501 (PMC5302693; doi:10.3390/proteomes2040501)
Supplement: Supplementary File 1 [file proteomes-02-00501-s001.pdf]

Supplementary Material

Table S1. Gene deletions that resulted in a 1.5-fold maximum decreases in abundance of fluorescently labelled TAG sequences. 133 deletion mutants had significantly different mean fluorescent signal in 56MESS exposed cultures relative to control ($p < 0.05$).

ORF	Gene	Fold-Change	*p*-value
YAL020C	ATS1	0.07	1.87E-03
YAL040C	CLN3	0.19	5.37E-02
YAL043C-A	KRE23	0.36	8.81E-02
YAL047C	SPC72	0.63	3.25E-02
YAL056W	GPE2	0.26	2.64E-02
YBL022C	PIM1	0.09	4.96E-02
YBL031W	SHE1	0.14	3.10E-02
YBL038W	MRPL16	0.06	5.74E-02
YBL079W	NUP170	0.19	5.89E-02
YBL099W	ATP1	0.27	2.12E-02
YBR189W	RPS9B	0.57	9.02E-03
YBR208C	"DUR1,2"	0.29	4.37E-02
YBR251W	MRPS5	0.55	5.14E-02
YBR268W	MRPL37	0.42	5.17E-02
YBR286W	APE3	0.31	1.11E-02
YBR291C	CTP1	0.08	6.47E-02
YCR027C	RSG1	0.09	4.77E-02
YCR028C	FEN2	0.49	3.85E-02
YCR053W	THR4	0.15	4.35E-02
YCR068W	CVT17	0.11	4.21E-02
YDL048C	STP4	0.16	3.98E-02
YDR075W	PPH3	0.22	2.22E-02
YDR080W	VPS41	0.14	1.08E-03
YDR140W	FYV9	0.31	6.24E-02
YDR147W	EKI1	0.58	1.64E-03
YDR175C	RSM24	0.54	9.94E-02
YDR225W	HTA1	0.51	7.14E-03
YDR226W	ADK1	0.41	1.09E-02
YDR227W	SIR4	0.56	3.43E-03
YDR234W	LYS4	0.42	4.29E-03
YDR245W	MNN10	0.41	3.18E-04
YDR253C	MET32	0.34	7.02E-02
YDR264C	AKR1	0.43	1.54E-03
YDR265W	PEX10	0.26	2.90E-02
YDR276C	PMP3	0.21	1.78E-02
YDR281C	PHM6	0.23	2.65E-02
YDR296W	MHR1	0.58	7.87E-05
YDR298C	ATP5	0.52	1.67E-03

Table S1. *Cont.*

ORF	Gene	Fold-Change	*p*-value
YDR300C	PRO1	0.38	1.13E-03
YDR335W	MSN5	0.23	9.76E-03
YDR385W	EFT2	0.11	2.46E-02
YDR389W	SAC7	0.10	7.49E-02
YEL053C	MAK10	0.57	4.74E-04
YER048C	CAJ1	0.48	1.53E-03
YER072W	VTC1	0.16	1.33E-04
YER116C	SLX8	0.62	2.12E-02
YFR040W	SAP155	0.26	4.72E-03
YGL035C	MIG1	0.50	6.64E-02
YGL148W	ARO2	0.16	6.88E-02
YGL153W	PEX14	0.42	1.25E-02
YGL212W	VAM7	0.05	5.85E-02
YGR007W	MUQ1	0.27	7.62E-02
YGR056W	RSC1	0.52	6.95E-02
YGR077C	PEX8	0.29	1.44E-02
YGR092W	DBF2	0.46	3.34E-02
YGR133W	PEX4	0.52	2.33E-02
YGR155W	CYS4	0.50	3.19E-02
YGR166W	KRE11	0.42	1.90E-02
YGR170W	PSD2	0.24	1.53E-03
YGR180C	RNR4	0.39	6.12E-03
YHL007C	STE20	0.22	6.06E-02
YHR039C	MSC7	0.54	6.81E-03
YHR103W	SBE22	0.48	2.38E-03
YHR104W	GRE3	0.27	1.75E-02
YHR108W	GGA2	0.26	4.43E-04
YHR129C	ARP1	0.35	4.05E-02
YHR204W	HTM1	0.58	2.82E-02
YIL030C	SSM4	0.59	2.95E-02
YIL033C	BCY1	0.35	4.97E-05
YIL052C	RPL34B	0.04	5.52E-03
YIL154C	IMP2'	0.01	6.65E-02
YJL024C	APS3	0.56	5.79E-02
YJL062W	LAS21	0.04	8.90E-02
YJL073W	JEM1	0.32	9.12E-03
YJL080C	SCP160	0.41	1.24E-02
YJL094C	KHA1	0.62	9.49E-02
YJL095W	BCK1	0.05	2.33E-02
YJL101C	GSH1	0.13	8.55E-02
YJL129C	TRK1	0.31	7.31E-03
YJL140W	RPB4	0.25	6.63E-02

Table S1. *Cont.*

ORF	Gene	Fold-Change	*p*-value
YJL165C	HAL5	0.05	6.86E-04
YJR040W	GEF1	0.50	3.12E-03
YJR117W	STE24	0.31	3.54E-02
YKL209C	STE6	0.53	2.80E-03
YKR006C	MRPL13	0.27	3.84E-02
YKR019C	IRS4	0.39	9.34E-02
YKR048C	NAP1	0.29	3.40E-02
YLL043W	FPS1	0.08	3.99E-03
YLR025W	SNF7	0.21	8.72E-02
YLR110C	CCW12	0.29	1.31E-02
YLR191W	PEX13	0.32	1.19E-02
YLR216C	CPR6	0.39	3.76E-02
YLR332W	MID2	0.19	1.52E-03
YLR360W	VPS38	0.26	3.71E-02
YLR393W	ATP10	0.10	7.41E-02
YLR443W	ECM7	0.52	1.95E-02
YML021C	UNG1	0.42	1.36E-02
YML067C	ERV41	0.35	2.56E-03
YML081C-A	ATP18	0.20	5.70E-02
YML106W	URA5	0.52	9.38E-02
YMR015C	ERG5	0.30	2.45E-02
YMR032W	HOF1	0.38	8.99E-02
YMR038C	LYS7	0.28	8.04E-02
YMR039C	SUB1	0.33	8.57E-03
YMR070W	MOT3	0.26	3.12E-02
YMR214W	SCJ1	0.08	1.69E-02
YNL054W	VAC7	0.003	3.26E-02
YNL183C	NPR1	0.08	2.47E-06
YNL197C	WHI3	0.10	2.47E-02
YNL214W	PEX17	0.45	1.19E-02
YNL257C	SIP3	0.35	2.19E-02
YNL283C	WSC2	0.24	8.69E-02
YNL288W	CAF40	0.59	1.91E-02
YNL291C	MID1	0.55	4.05E-02
YNL322C	KRE1	0.34	1.53E-02
YNL325C	FIG4	0.57	6.87E-02
YNR006W	VPS27	0.28	1.92E-03
YOL115W	TRF4	0.48	6.50E-02
YOR035C	SHE4	0.23	7.50E-02
YOR089C	VPS21	0.30	1.08E-02
YOR125C	CAT5	0.40	4.99E-02
YOR133W	EFT1	0.46	1.91E-03

Table S1. *Cont.*

ORF	Gene	Fold-Change	*p*-value
YOR216C	RUD3	0.02	4.04E-02
YOR297C	TIM18	0.15	1.35E-03
YOR360C	PDE2	0.54	4.18E-02
YOR371C	GPE1	0.42	4.94E-02
YPL002C	SNF8	0.40	8.80E-02
YPL078C	ATP4	0.50	8.04E-02
YPL120W	VPS30	0.05	2.89E-03
YPL161C	BEM4	0.23	3.11E-02
YPL178W	CBC2	0.30	3.75E-02
YPL226W	NEW1	0.20	3.63E-02
YPR164W	KIM3	0.53	1.68E-02

Figure S1. Distribution of gene function among 133 deletion mutants indicated as sensitive to 56MESS by microarray signal fold change (*i.e.*, ≥1.5-fold). Trafficking, protein handling, protein degradation and translation constitute a significant proportion of the functions identified using the deletion mutants.

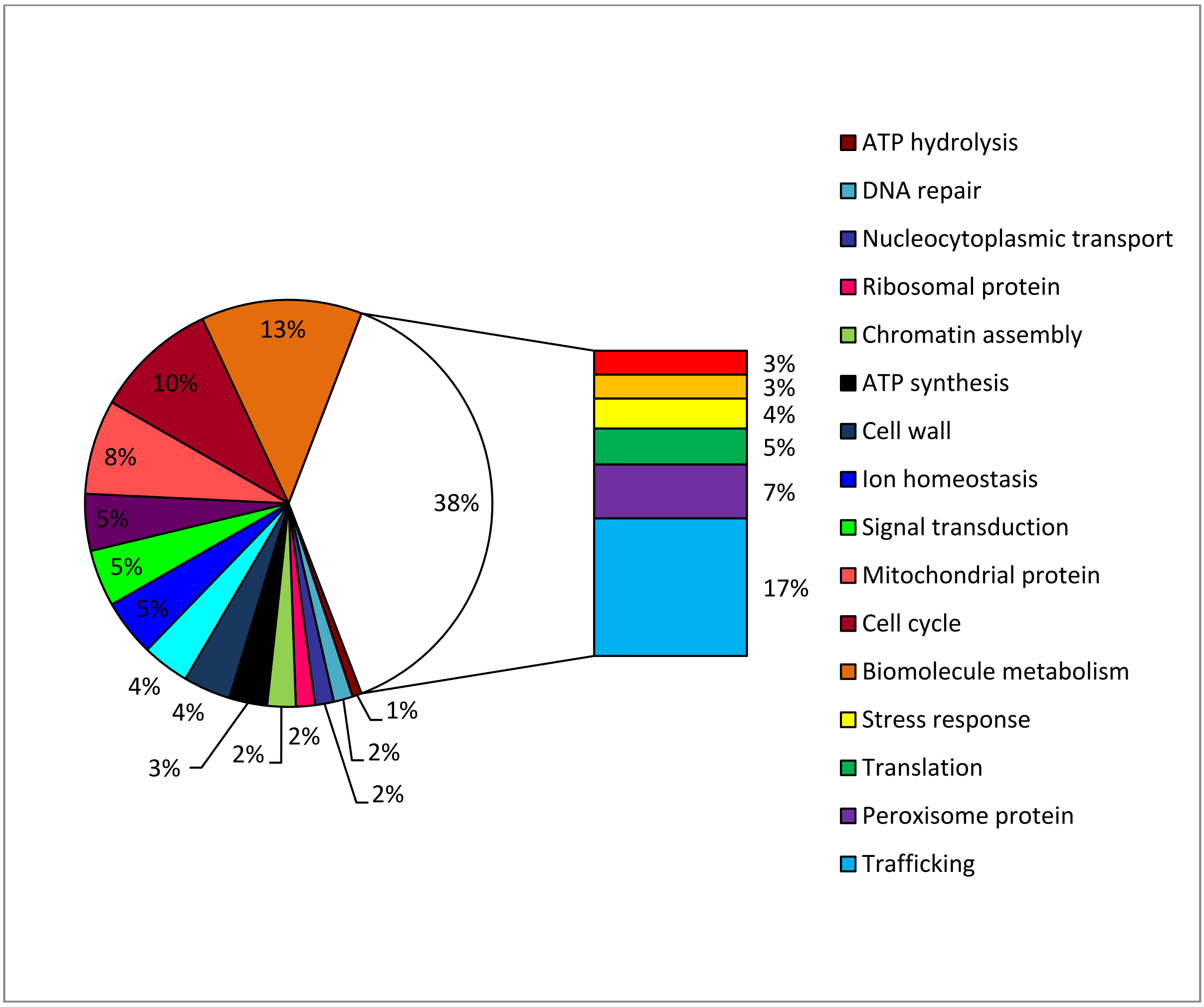

Figure S2. Kill curve using wildtype BY4743 yeast to determine comparable concentrations for 56MESS and cisplatin over a 2–8 h period. ⬩ Solvent; ▪ 1.5 mM 56MESS; ▴ 2 mM 56MESS; ▾ 1.0 mM cisplatin; ◆ 1.5 mM Cisplatin; ⊖ 2 mM cisplatin. Concentrations of cisplatin greater than 1.0 mM resulted in rapid cell death.

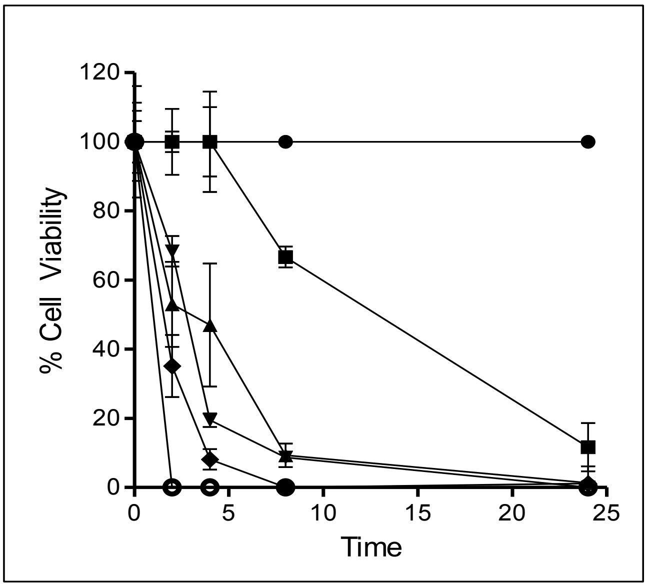

Table S2. Calculated theoretical molecular weights and isoelectric points of protein spots that show increased abundance following compound exposure. These spots undergo a minimum 2-fold change in fluorescent volume

Spot number	Isoelectric Point	Molecular Weight (kDa)	Fold Change
More abundant in cisplatin relative to Control			
1	4.9	17.3	2.0
2	4.7	17.2	2.2
3	4.7	13.3	2.3
4	4.9	27.2	2.4
5	4.5	13.3	2.6
6	6.1	36.1	3.4
7	4.6	21.7	9.9
8	4.5	23.3	11.5
More abundant in 56MESS relative to Control			
9	4.7	39.5	3.8
10	4.6	24.2	4.8
11	4.7	75.0	5.0
12	4.6	46.2	8.9
13	4.6	63.1	12.7
14	4.7	41.9	15.0
15	4.5	27.0	16.3
16	4.5	37.6	31.8

Table S3. Calculated theoretical molecular weights and isoelectric points of protein spots that show decreased abundance following compound exposure. These spots undergo a minimum 2-fold change in fluorescent volume.

Spot number	Isoelectric Point	Molecular Weight (kDa)	Fold Change
More abundant in control relative to cisplatin			
17	5.4	13.7	2.3
18	7.4	72.8	2.4
19	5.4	13.7	2.5
20	5.4	13.5	2.8
21	4.7	18.7	2.8
22	4.8	20.1	2.9
More abundant in control relative to 56MESS			
23	5.1	44.6	2.2
24	4.9	39.5	2.3
25	4.8	28.6	2.3
26	5.0	24.5	2.3
27	6.0	18.6	2.4
28	5.0	61.7	2.5
29	6.1	22.4	2.5
30	4.9	59.6	3.3
31	5.2	64.7	3.4
32	4.9	32.9	3.9
33	5.3	55.8	4.1
34	8.8	31.1	4.1
35	4.8	29.6	4.4
36	5.1	88.0	4.7
37	5.3	60.2	6.4
38	5.9	65.5	9.9

Table S4. Gene name and function summary from SGD of the 48 deletion mutants that were identified by microarray and confirmed by individual growth analysis. * indicate proteins that have molecular weight and pI characteristics that are comparable to a protein indicated in Tables S2 or S3.

ORF	Gene	Description (SGD)	Molecular Weight	Isoelectric Point	
YCR053W	THR4	Threonine synthase	57.5	5.4	*
YDR140W	MTQ2	S-adenosylmethionine-dependent methyltransferase	25.0	4.8	*
YMR070W	MOT3	Zinc finger transcription factor	54.4	9.2	
YIL154C	IMP2'	Ion homeostasis transcription factor	39.1	4.8	*
YMR015C	ERG5	Cytochrome P450 enzyme—sterol desaturase	61.3	7.9	
YML081C-A	ATP18	Subunit of mitochondrial F_1F_0 ATP synthase	6.7	10.5	
YDR226W	ADK1	Adenylate kinase for purine metabolism	24.3	6.4	*
YNL197C	WHI3	RNA binding protein that regulates cell fate	71.3	8.5	
YJL080C	SCP160	RNA-binding G protein effector of mating response	134.8	5.7	
YFR040W	SAP155	Required for Sit4p protein phosphatase complex	115.0	4.3	
YDR389W	SAC7	GTPase activating protein	73.6	10.4	
YMR032W	HOF1	SH3 domain-containing protein required for cytokinesis	76.2	9.9	
YGR092W	DBF2	Ser/Thr kinase involved in transcription and stress response	66.1	9.3	
YPL161C	BEM4	Protein involved in establishing cell polarity and buds	71.0	4.8	*
YAL020C	ATS1	Modifier of wobble nucleosides in tRNA, role in cell cycle	36.5	6.7	*
YNL322C	KRE1	Cell wall glycoprotein	32.2	9.2	*
YLR110C	CCW12	Cell wall mannoprotein	13.1	4.4	*
YBL022C	PIM1	ATP-dependent Lon protease	127.1	5.4	
YNL291C	MID1	N-glycosylated integral membrane protein, cation channel	61.8	4.2	*
YJR040W	GEF1	Voltage-gated chloride channel, Golgi	87.6	7.1	
YLR393W	ATP10	Assembly factor for mitochondrial F_1F_0 ATP synthase	32.1	10.4	*
YPL226W	NEW1	ATP binding cassette protein	134.3	5.7	
YBR291C	CTP1	Mitochondrial protein citrate transporter	32.2	10.4	*
YKR048C	NAP1	Histone chaperone	47.9	4.1	*
YDR335W	MSN5	Karypherin involved in nuclear import and export	142.1	5.1	
YER048C	CAJ1	Nuclear type II J heat shock protein	44.9	5.6	*
YJL129C	TRK1	Component of a potassium transport system	141.1	8.9	
YJR117W	STE24	Zinc metalloprotease	52.3	8.0	
YDR276C	PMP3	Small plasma membrane protein	6.1	4.0	
YJL165C	HAL5	Protein kinase	95.5	8.4	
YAL056W	GPB2	Multi-step regulator of cAMP-PKA signalling	98.7	7.2	
YJL095W	BCK1	MAPKKK in the protein kinase C signalling pathway	164.2	10.3	
YER072W	VTC1	Subunit of the vacuolar transporter complex	14.4	11.3	
YDR080W	VPS41	Vacuolar membrane protein, subunit of the HOPS complex	113.4	4.6	
YPL120W	VPS30	Subunit of phosphatidylinositol 3-kinase complexes I and II	63.3	4.8	*
YNR006W	VPS27	Endosomal protein required for recycling Golgi proteins	71.0	5.1	
YGL212W	VAM7	Vacuolar SNARE protein, trafficking	36.7	9.0	
YPL002C	SNF8	Component of the ESCRT-II complex	27.0	5.5	*

Table S4. *Cont.*

ORF	Gene	Description (SGD)	Molecular Weight	Isoelectric Point	
YOR216C	RUD3	Golgi matrix protein	56.1	4.7	*
YGR170W	PSD2	Phosphatidylserine decarboxylase of the Golgi and vacuolar membranes	130.1	7.9	
YDR245W	MNN10	Golgi mannosyltransferase complex protein	46.7	6.9	
YAL043C-A		Unknown, overlaps ERV46 (membrane fusion)	13.7	9.6	
YML067C	ERV41	Protein localised to COPII-coated vesicles	40.7	4.6	*
YCR068W	ATG15	Vacuolar lipase for intra-vacuolar lysis	58.4	5.3	*
YAL040C	CLN3	G1 cyclin involved in cell cycle progression	65.0	9.8	
YBR286W	APE3	Vacuolar aminopeptidase Y	60.1	5.0	*
YDR264C	AKR1	Palmitoyl transferase involved in protein palmitoylation	85.8	5.5	*
YNL183C	NPR1	Protein kinase	86.0	8.4	
